# Killing underweighted low viable newborn piglets: Which health parameters are appropriate to make a decision?

**DOI:** 10.1186/s40813-022-00265-y

**Published:** 2022-06-09

**Authors:** Lukas Geiping, Maria Hartmann, Lothar Kreienbrock, Elisabeth grosse Beilage

**Affiliations:** 1 Field Station for Epidemiology, University of Veterinary Medicine Hannover, Foundation, Bakum, Germany; 2 Institute for Biometry, Epidemiology and Information Processing (IBEI), University of Veterinary Medicine Hannover, Foundation, Hannover, Germany

**Keywords:** Pig, Neonate, Death, Mortality, Survival, Vitality, Welfare

## Abstract

**Background:**

The aim of this study was to estimate the mortality risk and associated factors within the first days of life for underweight or low-vital neonatal piglets. This risk estimation should start a discussion concerning the preconditions for timely killing of compromised newborn piglets to prevent unnecessary pain and suffering. In an observational study, various clinical and laboratory variables were examined in 529 piglets out of four farms. Body weight, crown-rump-length, rectal temperature, a 4-stage vitality score, an intrauterine-growth-retardation score, glucose, lactate, haemoglobin and immunocrit were assessed on the first day of life. Vitality was scored by three factors: movement, abdominal palpation, and colour of the skin. Afterwards the death of the piglets (by killing or spontaneously) was monitored until day 5 of age.

**Results:**

Body weight, rectal temperature and vitality score were significantly associated with probability of death. Piglets with rectal temperatures ≤ 37.5 °C, a body weight < 0.86 kg and impaired vitality scores were found to have the highest probability of death until day 5 of age.

**Conclusion:**

The clinical findings, identified by this model, allow a first estimation of mortality risk for newborn piglets within the first days of life. In a further step veterinarians, farmers and ethicians now need to clarify what probability of death should justifiy the killing of a newborn piglet.

**Supplementary Information:**

The online version contains supplementary material available at 10.1186/s40813-022-00265-y.

## Background

The management of low-viable newborn piglets is a distinct challenge for farmers. Reduced viability is associated with factors such as large litter size, low birth weight, longer duration of birth and reduced colostrum intake [1–4]. The requirement to manage low-viable piglets has increased as litters sizes have grown in the last decade [5, 6]. As litter sizes often exceed the number of functioning teats, management tools like split nursing to ensure colostrum intake just after birth [7] and the use of nurse sows to suckle redundant piglets [8] haven been developed. However, these tools cannot solve the fundamentally heightened mortality risk of underweighted or low-viable piglets. Even the return to breeding lines with lower litter sizes will merely reduce but not completely solve this problem as underweight and low viability do occur at any litter size [9]. Mortality rates among such piglets are significantly enhanced [10] and consequently some farmers tend to kill newborn pigs they expect to die within the first days of life. The farmers argue that killing low weight or low-viable piglets is enhancing animal welfare by effectively preventing these pigs from longer lasting suffering [11]. Animal welfare activists however retort that killing these pigs is not justified as it is primarily done for economic reasons [12]. Indeed, any decision regarding underweighted or low-viable newborn piglets is not easy to make as welfare and life of the piglet need to be protected [13]. While not killing a compromised piglet may affect welfare by exposing this individual piglet to a high chance of long-lasting suffering and death through starvation or crushing, killing compromised piglets avoids any welfare impairment but conflicts with the general protection of life [14]. Balancing those requirements in a way acceptable for the animal, the farmer as well as most of the society is far from easy. Therefore, the study described here is aimed at the identification of clinical variables appropriate for prediction of the death of underweight piglets aggregated until day 5 of age. These variables may become part of a guideline enabling farmers to arrive at a justified decision about killing a newborn piglet.

Based on previous literature, variables under study were selected [15] and tested for their capacity to predict the death of a piglet until day 5 of age. The selection was aimed at criteria easy to recognise for the farmer. Body weight, rectal temperature, crown-rump-length, a vitality score, and a score describing intra-uterine growth retardation (IUGR) were assessed to fulfil these criteria. Moreover, four blood measurements (glucose, lactate, haemoglobin, immunocrit), not easily available under on-farm conditions, were assessed to support the clinical data.

## Methods

### Herd characteristics

The study was conducted from May to October 2020 on three commercial pig producing farms and one research farm in Lower Saxony and North Rhine Westphalia with known and similar spontaneous mortality and killing rates. The sow herds consisted of three genetic lines (BundesHybridZuchtProgramm, Topigs, Danish Genetics) frequently used in Germany. Further farm details are shown in Table 1. In all herds, sows were inseminated with semen from Pietrain boars. In each herd, litters from three consecutive farrowing batches were included in the study. The study comprised a total of 529 piglets out of 99 litters (Table 1), ten piglets were excluded from further analysis due to missing data.

**Table 1:** Herd characteristics and number of sows and piglets included in the study

Herd Number	Sow herd size (number of sows)	Average life born piglets per litter (n)^a^	Average piglet mortality until weaning (%)	Sows included in the study (n)	Piglets included in the study (n)
1	200	16.2	16.4	22	124
2	1200	18.7	17.2	31	147
3	800	14.6	14.4	22	111
4	700	15.6	14.3	24	147

^a^Within 12 months before start of the study

### Sow management

Approximately one week before the expected farrowing date, the sows were moved to conventional farrowing pens equipped with a crate, fully slatted floors, and a heated creep area (about 30 °C). Ambient room temperature was set to 20 °C in the ventilated rooms. Farrowing supervision was performed by at least three caretakers per farm and took place between 6:00 am and 8:00 pm. Farrowing induction was not routinely used in any herd. All sows were vaccinated quarterly against porcine reproductive and respiratory syndrome virus, *Erysipelothrix rhusiopathiae* and parvovirus two weeks after farrowing.

### Piglet management

All litters were included in the study 12–24 h after birth (day 1) before cross-fostering and litter equalization was done.

All farms used nurse sows and additional feeders to ensure adequate milk and feed supply of the newborn piglets. Tail docking and teeth grinding were performed on day 2 and all piglets were treated with an iron preparation on day 3 or 4. At the same time male piglets were surgically castrated without anaesthesia but with analgesia (intramuscular injection of 0.4 mg/kg meloxicam (Metacam® 5 mg/ml, Boehringer Ingelheim, Ingelheim, Germany)) according to the German Animal Protection Act valid at that time [16].

### Data collection

For identification, all piglets were marked with individually numbered ear tags on day 1.

### Clinical variables evaluated at day 1

Piglets with body weight (BW) at birth ≤ 1.0 kg (low weight group, LW) were compared to piglets with a body weight > 1.0 kg (normal weight group, NW). While all piglets from a selected litter weighing ≤ 1.0 kg were included in the LW, the number of piglets included in the NW was restricted to two piglets per litter due to limited capacity. In total, the LW comprises 328 piglets and the NW 191 piglets. For further analysis, LW and NW were subdivided based on whether they were dead (killed by the investigator/farmer or spontaneously died) or alive at day 5 (low weight group alive (LWA) and low weight group dead (LWD), normal weight group alive (NWA) and normal weight group dead (NWD)).

The data recorded on day 1 also comprised the sex, the rectal temperature and the crown-rump length (CRL), defined as the length from the occiput to the base of the tail. The vitality was assessed based on a score from 0 to 3 on day 1 to day 5 (Table 2). A score of 0 was given if the piglets showed no signs of reduced vitality. A score of 1 was given for piglets showing one sign of moderately reduced vitality, score 2 was recorded when a piglet showed two signs of moderately reduced vitality and score 3 when one or more signs of severely reduced vitality were diagnosed. Piglets with a score of 3 were categorized as non-viable and were killed immediately by the farmer by blunt force trauma and subsequent bleeding or by the investigator by intravenous injection of a lethal dose of Pentobarbital (Release® 500 mg/ml, WDT, Garbsen, Germany, dosage: 450 mg/5 kg BW).

**Table 2:** Clinical variables evaluated in piglets at day 1

Clinical variable	Measuring unit				
Sex	Female/male				
Body weight (BW)	Kilogram (kg)				
Crown-rump length (CRL)	Meter (m)				
Rectal temperature (RT)	Centigrade (°C)				
Vitality score (VS)	Vitality Criteria	Unaffected	Mild to moderate	Severe	
	Colour of the skin	Pink	Pale	Cyanotic	
	Movement	Stable	Unstable	Unable to stand	
	Abdominal palpation	Well filled	Moderately filled	Empty	
	Overall vitality score	VS 0	VS 1	VS 2	VS 3
		No signs of reduced vitality	1 sign of moderately reduced vitality	> 1 sign of moderately reduced vitality	≥ 1 sign of severely reduced vitality
Intrauterine-growth retardation (IUGR) score	Score 0 (no IUGR),1 (mild IUGR), 2 (severe IUGR) based on the criteria				
	Dolphin like head (yes/no)				
	Bulging eyes (yes/no)				
	Wrinkles around nose and eyes (yes/no)				

The piglets were also scored for IUGR based on the scheme evaluated by [17, 18]. A score of 1 (mild IUGR) was given if the piglet showed at least one sign of IUGR and a score of 2 (severe IUGR) when more than one sign was proven (Table 2).

### Blood measurements evaluated at day 1

Blood was collected from all piglets on day 1 by punctuation of the *V. cava cranialis* for laboratory measurements regarding the survival or death of neonatal piglets. The sampling was done by collecting 0.5 mL blood and by using a 23 G needle. The sampling took in general less than 20 s of time. A part of the blood was immediately analysed on-farm using two different hand-held devices. The remaining blood was transferred into 1 ml Eppendorf tubes (Eppendorf AG®, Hamburg, Germany) and stored at 8 °C until further investigation in the laboratory. With two Accutrend® Plus devices (Roche Diagnostics Deutschland GmbH, Mannheim, Germany), glucose and lactate concentrations were measured in whole blood as previously described [19, 20]. Both devices were checked with control solutions provided by the manufacturer (Accutrend® Control G2; BM-Control G2, Roche Diagnostics Deutschland GmbH, Mannheim, Germany) before testing each batch. The range for glucose concentrations measurable with this device ranges from 20 to 600 mg/dl. Values below this range were classified as 19 mg/dl. Lactate concentration was measured from 0.8 to 22 mmol/L; higher values were classified as 23 mmol/L. The Hemocue® Hb 201 Analyzer (Hitado GmbH, Möhnesee, Germany) was used to measure haemoglobin concentration [21]. The measuring scale runs from zero to 15.9 mmol/L.

To assess the amount of colostrum the piglets have ingested, the immunocrit was measured, as previously described [22]. The blood, stored as described above, was centrifuged at 2000 rpm for 10 min and serum was separated. 50 µl of serum was mixed with 50 µl of 40% ammonium sulfate solution in a 0.5 ml Eppendorf tube and left to precipitate for two minutes. The sample was then centrifuged at 13,000 g for five minutes in hematocrit tubes. The immunocrit was assessed as a dimensionless ratio of the length of the white precipitate and the whole length of the solution.

### Killing piglets

Based on the precautions which are set for animal trials by the German federal institutes (LAVES and LANUV) it was determined beforehand that piglets with a vitality of score 3 (Table 2), anomalies or lacerations had to be killed immediately by the investigator or farmer. All farmers participate voluntarily in this study, however confirmed their willingness to suspend their routinely used killing procedures for all piglets included in the study and follow this specific protocol.

### Post-mortem examination

All piglets included in the study that died spontaneously or had to be killed until day 5 were weighed and collected for a standardized post-mortem examination (Table 3). The cause of death was recorded (crushing, starvation, infection, anomalies, other) as well as whether the piglet had been killed or died spontaneously. The diagnosis “crushing” was assigned when the piglet had broken bones or when typical internal or external lesions or bleedings were detected. “Starvation” was diagnosed when the piglet was emaciated, and ribs or other prominent bones were easily visible. The diagnosis “infection” included all piglets showing signs of enteritis, pneumonia or arthritis. Under “anomaly” splay legs, blind anus and other congenital malformations incompatible with survival have been summarized. The diagnosis “other” was recorded when the cause of death could not be ascertained.

**Table 3:** Post-mortem variables evaluated in piglets that died spontaneously or have been killed during the study period

Variables	Outcome
Date of death	Study day
Death	Spontaneous Killed
Cause of death/reason for killing	Crushing Starvation Weak/non-viable Infection Laceration/anomaly Other
Stomach milk content	Yes/no
Body weight at killing/when found dead	Kilogram

### Sample size calculation and statistical analyses

Calculation of the investigation's sample size was performed using NCSS-PASS-software. To control for multivariable influence within the logistic regression the concept of Hsieh et al. [23] was applied to identify Odds Ratios of 2 with a multiple correlation of factors of 0.7, which implies an overall sample size of 542. In addition, for quantitative data a Wilcoxon rank-sum test comparing two groups with a delta = 1.1 and sigma = 3.2 was applied yielding in an entire sample size of 564 piglets. For both calculations the type I error was set to 5% and the power to 80%. Death (by killing or spontaneously) of a newborn piglet until day 5 of age was analysed using a linear logistic regression analysis. Herds and batches per herd were combined to one factor and included as fixed effect in this model. Body weight (day 1) was transferred into three categories comprising piglets < 0.86 kg (BW 1), 0.86 to 1.00 kg (BW 2), and > 1.00 kg (BW 3). Rectal temperature was differentiated by two groups with ≤ 37.5 °C (RT 1) and > 37.5 °C (RT 2).). For the discussion of the results outcome death was distinguished in two groups of killed and spontaneously death animals to perform sensitivity analyses due to the outcome (see Additional files 1 and 2: Tables S1 and S2). An extended model was used including immunocrit and glucose to study if laboratory measures improve the model fit (see Additional file 3: Table S3).

Statistical significance was assumed for *p* < 0.05. Due to the explorative nature of the study, no adjustment for multiple comparisons was taken into account. All statistical evaluations were performed with SAS®, version 9.4 TS level 1M5 (SAS Institute Inc., Cary, NC, United States) using LOGISTIC and GLIMMIX as model procedures.

## Results

**Fig. 1:**
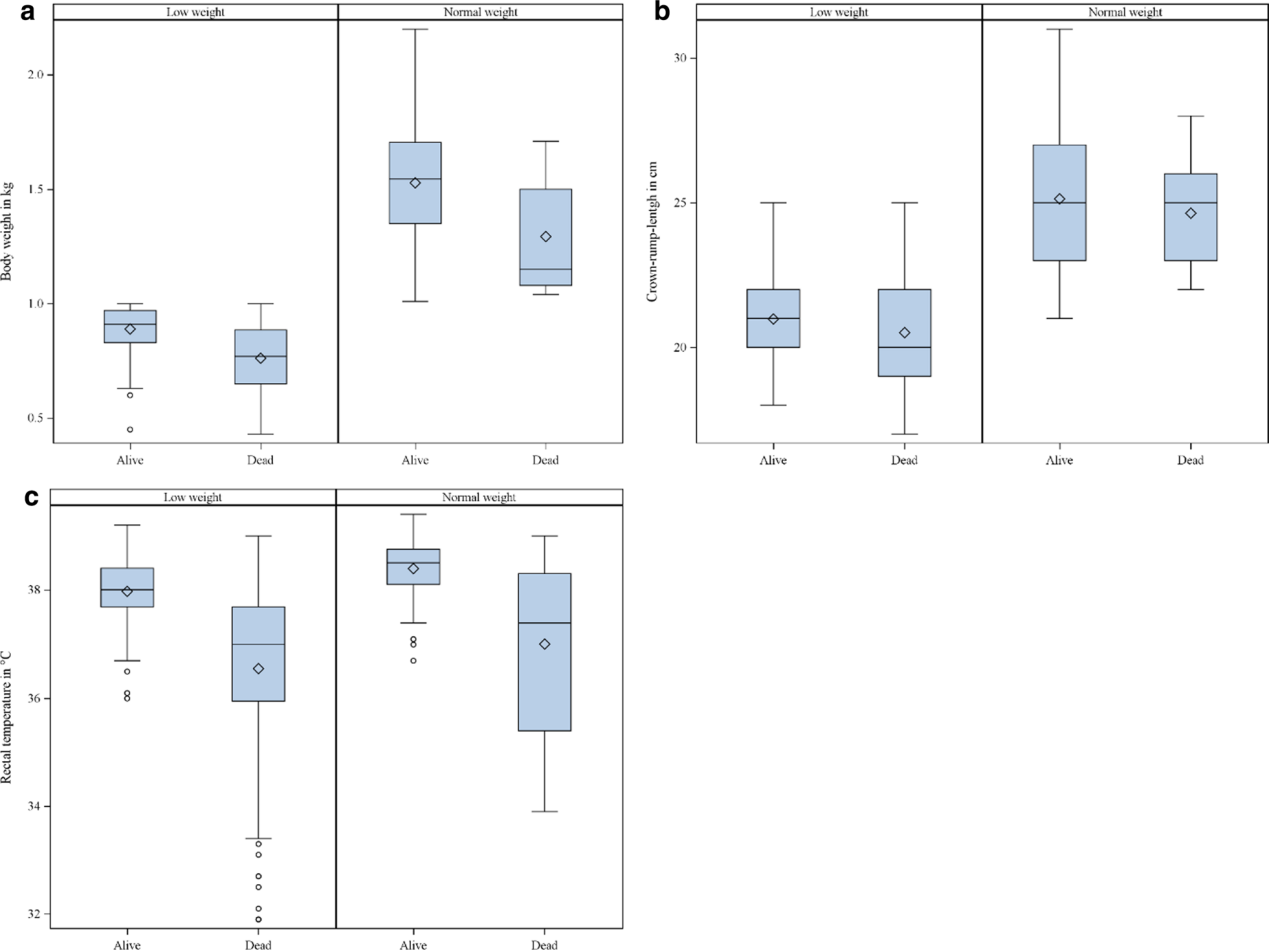
**a**–**c** Descriptive statistics of metric clinical variables in newborn piglets of the low weight group and normal weight group dying or surviving until day 5 of age

**Fig. 2:**
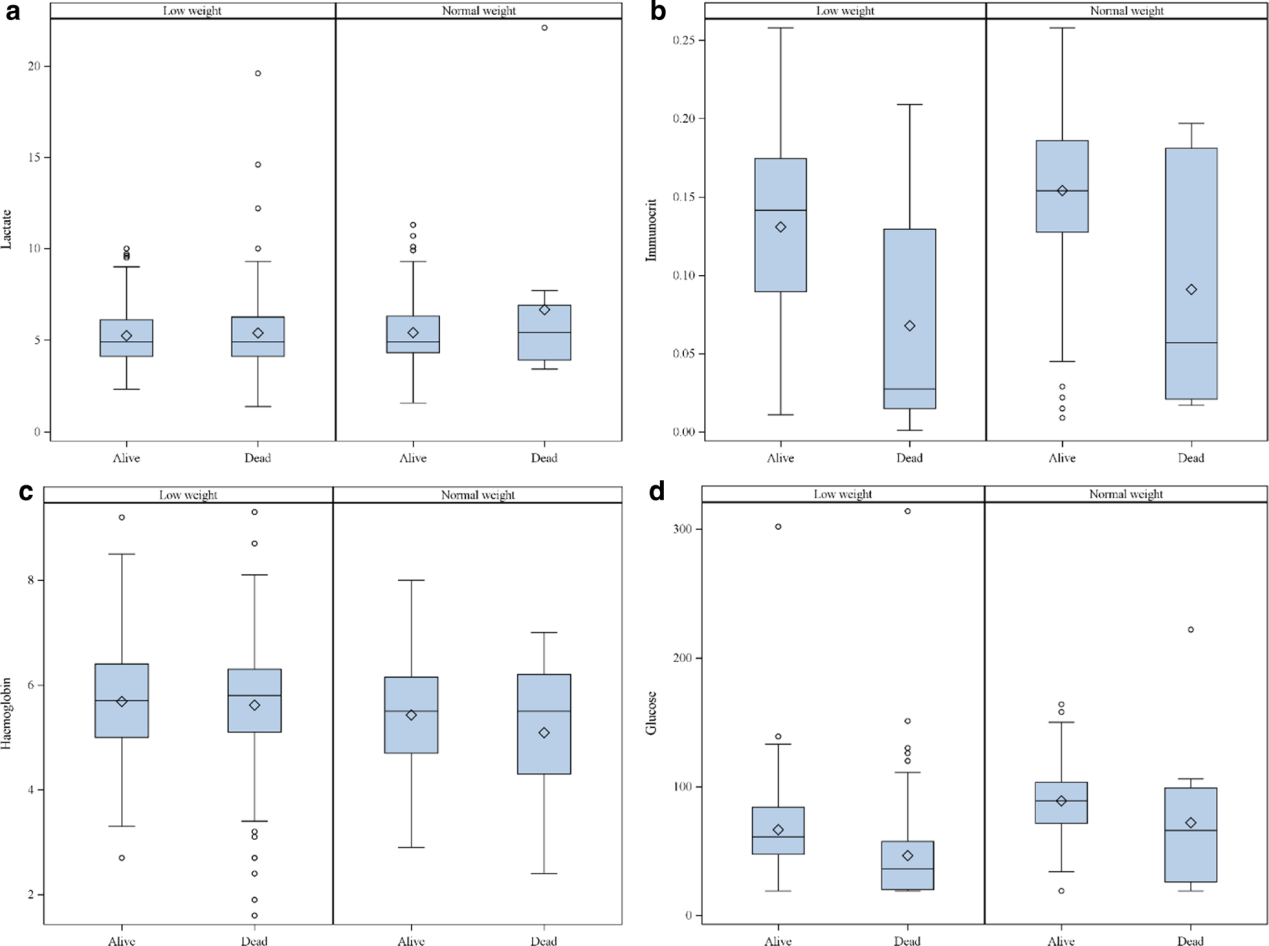
**a**–**d** Descriptive statistics of metric laboratory variables in newborn piglets of the low weight group and normal weight group dying or surviving until day 5 of age

Birth weight, crown-rump-length and rectal temperature showed higher values in surviving than in dying piglets (Fig. 1a–c). A higher vitality score or a higher IUGR score was also associated to a higher chance of mortality until day 5 (Table 4). The same effects were found in the laboratory measurements glucose and immunocrit (Fig. 2a–d). Both variables showed statistically significant differences in the univariable model (*p* < 0.0001) but not in the multivariable model (immunocrit *p* = 0.0556; glucose *p* = 0.1382) (see Additional file 3: Table S3). A higher vitality score or a higher IUGR score was also associated to a higher chance of mortality until day 5 (Table 4).

**Table 4:** Mortality by vitality score (VS) and intrauterine growth retardation (IUGR) in LW (low weight) and NW (normal weight) newborn piglets dying or surviving until day 5 of age

Category	LW		NW	
	Number of piglets per category	Percentage of dead piglets per category	Number of piglets per category	Percentage of dead piglets per category
Vitality score (VS)				
0	146	16.4	180	7.9
1	127	46.5	9	33.3
2	29	93.1	0	0
3	26	100	0	0
Intrauterine growth retardation (IUGR)				
0	124	22.6	187	4.8
1	133	36.8	3	33.3
2	71	83.1	1	100

**Fig. 3:**
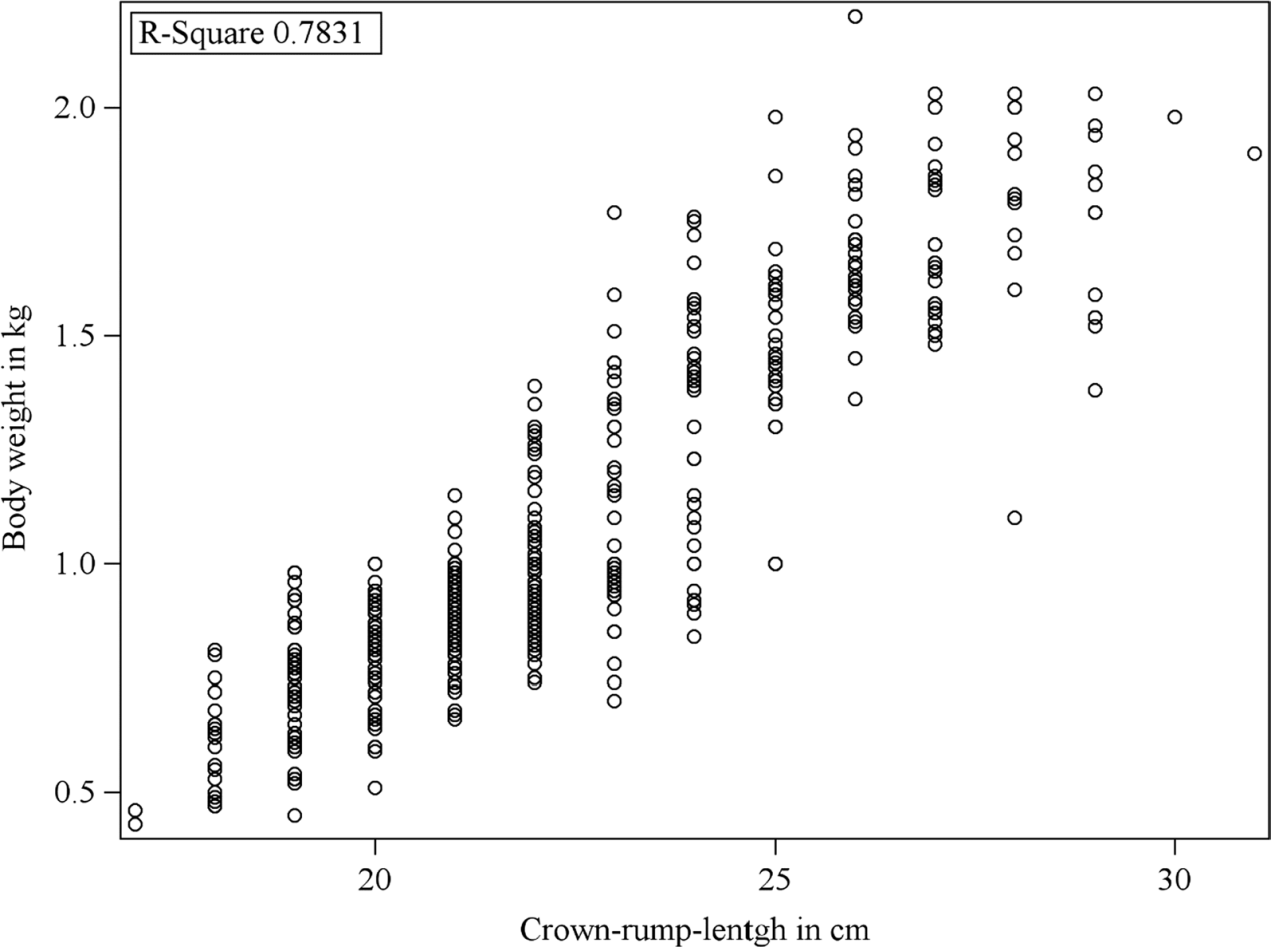
Pearson correlation between bodyweight and crown-rump length at day 1 of age

Crown-rump length was highly correlated with body weight at day 1 (Fig. 3) and, therefore, not considered for the final model. Sex was not significantly associated to mortality, but male piglets tend to have a higher mortality rate (*p* = 0.0680) (Table 5).

**Table 5:** Logistic regression model for clinical variables related to *killing or spontaneously death* of newborn piglets dying until day 5 of age

Risk categories	Alive		Dead		Univariable model				Multivariable model			
	n	%	n	%	OR	95%-CI		*p*	OR	95%-CI		*p*^b^
						Low	Up			Low	Up	
Total	372	71.68	147	28.32	x	x	x	x	x	x	x	x
Herd/group (*p* = 0.0033) ^a^												
11(ref)	26	56.52	20	43.48	1	x	x	x	1	x	x	x
12	27	65.85	14	34.15	0.674	0.282	1.609	0.3741	0.229	0.053	0.984	0.4722
13	19	51.35	18	48.65	1.232	0.516	2.937	0.6386	0.618	0.145	2.636	0.2897
21	33	73.33	12	26.67	0.473	0.196	1.141	0.0956	0.281	0.074	1.071	0.7145
22	25	60.98	16	39.02	0.832	0.353	1.959	0.6738	0.845	0.239	2.989	**0.0373**
23	38	74.51	13	25.49	0.445	0.189	1.049	0.0643	0.326	0.086	1.232	0.9287
31	26	83.87	5	16.13	0.250	0.082	0.767	**0.0153**	0.083	0.014	0.479	**0.0451**
32	35	87.50	5	12.50	0.186	0.062	0.560	**0.0028**	0.212	0.048	0.933	0.4034
33	38	95.00	2	5.00	0.068	0.015	0.318	**0.0006**	0.102	0.017	0.627	0.1074
41	43	82.69	9	17.31	0.272	0.108	0.686	**0.0058**	0.195	0.055	0.687	0.2282
42	28	66.67	14	33.33	0.650	0.273	1.547	0.3301	1.523	0.430	5.394	**0.0005**
43	34	64.15	19	35.85	0.726	0.323	1.632	0.4390	0.401	0.117	1.369	0.6833
Body weight (*p* = 0.0418) ^a^												
≤ 0.86 kg	69	41.07	99	58.93	23.475	11.870	46.424	** < .0001**	3.715	1.103	12.507	0.1221
0.86–1 kg	123	76.88	37	23.13	4.922	2.417	10.023	** < .0001**	2.973	1.238	7.140	0.1885
> 1 kg (ref)	180	94.24	11	5.76	1	x	x	x	1	x	x	x
Vitality score (*p* ≤ .0001) ^a^												
0 (ref)	296	90.80	30	9.20	1	x	x	x	1	x	x	x
1	74	54.41	62	45.59	8.265	4.989	13.694	** < .0001**	3.325	1.589	6.958	0.0710
2	2	3.51	55	96.49	271.333	63.013	1168.351	** < .0001**	61.358	10.609	354.867	** < .0001**
Intrauterine growth retardation score (*p* = 0.1765) ^a^												
0 (ref)	274	88.10	37	11.90	1	x	x	x	1	x	x	x
1	86	63.24	50	36.76	4.305	2.640	7.022	** < .0001**	1.582	0.656	3.814	0.6534
2	12	16.67	60	83.33	37.027	18.231	75.201	** < .0001**	3.412	0.929	12.538	0.0678
Rectal temperature (*p* = 0.0003) ^a^												
≤ 37.5 °C	48	32.21	101	67.79	14.818	9.336	23.519	** < .0001**	3.372	1.745	6.517	**0.0003**
> 37.5 °C (ref)	324	87.57	46	12.43	1	x	x	x	1	x	x	x
Sex (*p* = 0.0680)^a^												
Female (ref)	187	73.91	66	26.09	1	x	x	x	1	x	x	
Male	185	69.55	81	30.45	1.241	0.846	1.820	0.2704	1.712	0.961	3.049	0.0680

Due to missing values vitality scores 2 and 3 were aggregated to one category for modelling purposes

ref, reference category; OR, odds ratio estimate; CI, confidence limits; p, level attained for the statistical test associated

^a^General *p* value Wald’s Chi^2^-test (*p* < 0.05 are marked in bold)

^b^*p* value Wald’s Chi^2^-test (*p* < 0.05 are marked in bold) to the reference category

There were significant differences between the groups defined by herds and farrowing batches (*p* = 0.0033) (Table 5).

In the multivariable logistic regression model, a bodyweight < 1.0 kg (*p* = 0.0418), a vitality score 1 or 2 (*p* ≤ 0.0001) and a rectal temperature ≤ 37.5 °C (*p* = 0.0003) were identified as associated with the event of death of a newborn piglet until day 5 (Table 5).

The difference between piglets that were killed and piglets that died spontaneously is shown in the Additional files 1 and 2: Tables S1 and S2. The regression model for killed piglets showed that only the variable vitality remains statistically significant (*p* ≤ 0.0001). Piglets that died spontaneously had a statistically significant lower rectal temperature (*p* = 0.0002) or a higher vitality score (*p* = 0.0063). Body weight was not identified as associated (*p* = 0.0598). The herd/group effect was also statistically significant (*p* = 0.0294).

Based on the adjusted Odds ratios of the results of the logistic regression model, prognostic values for the probability of a newborn piglet to die until day 5 of age were derived, considering the different combinations of body weight, rectal temperature and vitality score (Table 6). A probability of 94%, 92% and 81% to die until day 5 was determined for the combination rectal temperature ≤ 37.5 °C and a vitality score 2 in the body weight groups < 0.86 kg, 0.86–1.0 kg and > 1.0 kg (Table 6).

**Table 6:** Predicted probability for the death of a newborn piglet until day 5 of age assessed by clinical parameters identified from the multivariable logistic regression model (see Table 5)

Weight group	Vitality score	Rectal temperature	Probability of death
< 0.86 kg	0	≤ 37.5 °C	0.24
< 0.86 kg	0	> 37.5 °C	0.08
< 0.86 kg	1	≤ 37.5 °C	0.49
< 0.86 kg	1	> 37.5 °C	0.21
< 0.86 kg	2	≤ 37.5 °C	0.94
< 0.86 kg	2	> 37.5 °C	0.81
0.86–1.0 kg	0	≤ 37.5 °C	0.20
0.86–1.0 kg	0	> 37.5 °C	0.06
0.86–1.0 kg	1	≤ 37.5 °C	0.43
0.86–1.0 kg	1	> 37.5 °C	0.17
0.86–1.0 kg	2	≤ 37.5 °C	0.92
0.86–1.0 kg	2	> 37.5 °C	0.77
> 1.0 kg	0	≤ 37.5 °C	0.08
> 1.0 kg	0	> 37.5 °C	0.02
> 1.0 kg	1	≤ 37.5 °C	0.21
> 1.0 kg	1	> 37.5 °C	0.07
> 1.0 kg	2	≤ 37.5 °C	0.81
> 1.0 kg	2	> 37.5 °C	0.53

Mortality in the LW was 41.5% (n = 136) and in the NW 5.8% (n = 11). In LW 18.4% and 27.2% in NW were killed as they showed severely reduced vitality (VS 3). The other piglets died spontaneously, 32.4% (LW) and 18.2% (NW) by crushing, 39.7% (LW) and 27.2% (NW) due to starvation. In 89.0% (LW) and 72.7% (NW) of the dead piglets the stomach was found empty. 46 piglets died or had to be killed on day 1 (31%), 57 piglets on day 2 (39%), 28 on day 3 (19%), 13 on day 4 (9%), and 3 on day 5 (2%).

## Discussion

Care of newborn piglets is one of the main animal welfare issues in piglet-producing farms [6, 14, 24]. While most studies are focused on improvement of piglet survivability [25–27] only a very few publications discuss the conditions of compromised newborn piglets with low chance for survival [2, 24, 28, 29]. Based on the authors’ knowledge, there are currently no publications that describe an approach to classify the probability of death of these animals. As the options for intensive care are limited under the standard conditions of piglet production, some farmers choose a pragmatic approach by killing newborn piglets they expect having less economical value [11]. However, this practice is neither in accordance with the different animal welfare acts nor accepted by the majority of people in Europe [30]. The opposite, also practiced on some farms, ignoring compromised newborn piglets and let them die without or only a little intervention is also not acceptable, as this may result in avoidable pain and/or suffer [31, 32]. A first step into a way out of this dilemma might be found in a validated assessment scheme facilitating an estimation of the probability of death of a newborn piglet until five days of age. This scheme is aimed at giving farmers a clear guideline leading to a comprehensive decision about care or killing of a compromised newborn piglet.

Based on literature [10, 20, 33] a set of clinical and laboratory measurements associated with increased mortality in newborn piglets was selected. The selection was focussed on clinical variables that can be assessed easily by farmers and resulted in body weight, rectal temperature, crown-rump-length, a vitality score and an IUGR score. As clinical variables are often estimated being “subjective” compared to “objective” laboratory parameter, additionally a set of laboratory measurements was selected, expected to be appropriate in predicting the death of a newborn piglet [34–36].

In general, the clinical variables can be easily and quickly measured by the farmer. The vitality score [37] is based on findings assessing the colour of the skin, the movement and the filling of the abdomen (Table 2). With three items and findings that need to be assigned to one of only four categories (unaffected, mild, moderate, and severe) the vitality score is estimated to be appropriate for the application by farmers. Further studies focussed on the inter- and intra-observer repeatability of the vitality scoring may help evaluating the training effort.

Rectal temperature is proved to be associated to mortality and is a clear indicator for hypothermia [9].

The filling of the abdomen is indicating the colostrum/milk intake. Piglets are born without immunoglobulins and are highly dependent on the early intake of colostrum [3]. The measurement of milk intake by abdominal palpation as a part of the vitality score and the measurement of the immunocrit in this study clearly underlined the importance of colostrum and milk intake on neonatal survival (for the influence of the immunocrit in the logistic regression model: see Additional file 1: Table S1)

Since piglets are born with no brown adipose tissue, they are only able to maintain their body temperature through liver and muscle glycogen. These reserves only last for 16–24 h [38]. Blood glucose levels are described in various studies as highly significant to survival [34, 35]. However, the increase of glucose in agony or through catecholamine release in stressful situations aggravates the interpretation [4, 39]. The univariate model showed a significant association between mortality and glucose, but the multivariable model showed no significant association (see Additional file 3: Table S3).

Hypoxia during prolonged births or birth in the last third of parturition leads to an increase of lactate [40, 41]. Higher haemoglobin levels at birth make piglets less prone to hypoxia due to the higher oxygen carrying capacity [42]. No association to survival was seen for both variables in this study. This may be caused by the inclusion into the study 12–16 h after birth. Piglets suffering from hypoxia could already have died.

The farmers were participating voluntarily in the study. The study herds were managed according to the rules of good farming practice, ensuring standard housing conditions and extensive care of the pigs. The study herds were carefully selected to exclude an influence of poor housing or management on the results. As in other studies [43, 44], an effect of herd and batch was confirmed but body weight, rectal temperature and vitality score remained significantly associated to mortality. Therefore, one can assume that these variables are applicable universally and that the differences between farms and batches cannot rule out the effect of these variables. The study was focussed on the identification of variables helping a farmer to identify a compromised piglet and find an appropriate solution for this individual animal. However, it was not an objective of this study to identify the specific reasons for low body weight, low rectal temperature or reduced viability [2, 4, 10, 14, 17].

Splitting the data between piglets that died spontaneously and piglets that were killed modify the results in an expected direction. In the group of killed piglets, only the vitality score remained significant in the regression model. This result is not unexpected as the vitality score was one of the variables the animal welfare authorities had determined to require immediate killing. Abnormalities and lacerations, the other findings that require immediate killing have not been observed in this study. In the group of piglets that died spontaneously only the variable rectal temperature remained as statistically significant. This effect on one hand is likely induced by the reduced statistical power in this group and on the other hand on a higher vitality in this group. For a better understanding of all effects that might influence mortality it would have been advantageous to completely resign on killing piglets, however such study design is not in accordance with German animal welfare laws and the precautions which are set for animal trials by the German federal institutes (LAVES and LANUV).

Higher mortality of male piglets, which is probably based on a different body composition [29] or influenced by surgical castration, was expected but could not be confirmed. Surgical castration might be a risk but, in this study, the majority of piglets died before they were castrated on day three or four of age. In further studies, the effect of castration should be ruled out by monitoring entire boars.

Blood sampling was also suspected to have intervened with the outcome of the study by affecting animals in a negative way. However, the procedure was done with consideration by a well-trained investigator using very fine needles and collecting only a very small amount of blood (0.5 ml). A further study without blood sampling could confirm the results presented in this study and diminish the potential influence of the procedure on the vitality of newborn piglets.

## Conclusions

The results of this study show that the death of a newborn piglet can be predicted by a simple clinical score applicable for trained veterinarians and farmers. Nonetheless, it needs to be emphasized that the proposed method is not yet ready to be used in practice because of the exploratory nature of this study. To generalize the results of this study a representative sample of farms need to be included as well as different observers. Nevertheless, the authors think that this study can be used as a basis for further investigations. In conclusion veterinarians, farmers and particularly ethicists will need to clarify in an extensive process what probability of death will justify the killing of a newborn piglet.

## Supplementary Information

**Additional file 1:** **Table 1**. Logistic regression model for clinical variables related to piglets that were **killed** until day 5 of age. Ref: reference category, OR: Odds Ratio Estimate, CI: confidence limits. p: level attained for the statistical test associated (^a^General p-value Wald’s Chi²-Test; ^b^p-value Wald’s Chi²-Test to the reference category).

**Additional file 2:** **Table 2**. Logistic regression model for clinical variables related to piglets that **spontaneously died** until day 5 of age. Ref: reference category, OR: Odds Ratio Estimate, CI: confidence limits. p: level attained for the statistical test associated (^a^General p-value Wald’s Chi²-Test; ^b^p-value Wald’s Chi²-Test to the reference category).

**Additional file 3:** **Table 3**. Logistic regression model for clinical and laboratory variables related to killing or death of newborn piglets dying until day 5 of age. Ref: reference category, OR: Odds Ratio Estimate, CI: confidence limits. p: level attained for the statistical test associated (^a^General p-value Wald’s Chi²-Test; ^b^p-value Wald’s Chi²-Test to the reference category).

## Data Availability

Please contact the corresponding author for data requests. This Open Access publication was funded by the Deutsche Forschungsgemeinschaft (DFG, German Research Foundation) within the programme LE 824/10-1 "Open Access Publication Costs" and the University of Veterinary Medicine Hannover, Foundation.
