# Supplementary material for: Killing underweighted low viable newborn piglets: Which health parameters are appropriate to make a decision?
Source: Porcine Health Manag. 2022 Jun 9;8:25. doi: 10.1186/s40813-022-00265-y (PMC9178864; doi:10.1186/s40813-022-00265-y)
Supplement: Supplementary file 1 — Additional file 1: Table 1. Logistic regression model for clinical variables related to piglets that were killed until day 5 of age. Ref: reference category, OR: Odds Ratio Estimate, CI: confidence limits. p: level attained for the statistical test associated (aGeneral p-value Wald’s Chi²-Test; bp-value Wald’s Chi²-Test to the reference category). [file 40813_2022_265_MOESM1_ESM.docx]

Additional file table 1: Logistic regression model for clinical variables related to piglets that were **killed** until day 5 of age. ref: reference category, OR: Odds Ratio Estimate, CI: confidence limits. p: level attained for the statistical test associated

| Risk categories | alive | | killing | | univariable model | | | | multivariable model | | | |
| --- | --- | --- | --- | --- | --- | --- | --- | --- | --- | --- | --- | --- |
|  | n | % | n | % | OR | 95%-CI | | p | OR | 95%-CI | | p^b^ |
|  |  |  |  |  |  | low | up |  |  | low | up |  |
| Total | 372 |  | 73 |  | x |  |  | x | x |  |  | x |
| Herd/Group (p=0.1374)^a^ | | | | | | | | | | | | |
| 11(ref) | 26 | 81.25 | 6 | 18.75 | 1 | x | x | x | 1 | x | x | x |
| 12 | 27 | 72.97 | 10 | 27.03 | 1.605 | 0.510 | 5.050 | 0.4187 | 0.708 | 0.070 | 7.173 | 0.9398 |
| 13 | 19 | 59.38 | 13 | 40.63 | 2.965 | 0.954 | 9.214 | 0.0603 | 1.681 | 0.180 | 15.671 | 0.9151 |
| 21 | 33 | 82.50 | 7 | 17.50 | 0.919 | 0.275 | 3.069 | 0.8910 | 0.363 | 0.030 | 4.428 | 0.9589 |
| 22 | 25 | 69.44 | 11 | 30.56 | 1.907 | 0.612 | 5.939 | 0.2656 | 4.941 | 0.584 | 41.801 | 0.8845 |
| 23 | 38 | 86.36 | 6 | 13.64 | 0.684 | 0.199 | 2.356 | 0.5475 | 0.540 | 0.063 | 4.646 | 0.9476 |
| 31 | 26 | 96.30 | 1 | 3.70 | 0.167 | 0.019 | 1.483 | 0.1081 | 0.020 | <0.001 | 0.691 | 0.9578 |
| 32 | 35 | 100.00 | 0 | 0 | x | x | x | x | x | x | x | x |
| 33 | 38 | 100.00 | 0 | 0 | x | x | x | x | x | x | x | x |
| 41 | 43 | 93.48 | 3 | 6.52 | 0.302 | 0.070 | 1.314 | 0.1105 | 0.314 | 0.039 | 2.539 | 0.9631 |
| 42 | 28 | 87.50 | 4 | 12.50 | 0.619 | 0.157 | 2.444 | 0.4937 | 1.748 | 0.164 | 18.600 | 0.9140 |
| 43 | 34 | 73.91 | 12 | 26.09 | 1.529 | 0.507 | 4.617 | 0.4512 | 1.176 | 0.155 | 8.924 | 0.9253 |
| Body Weight (p=0.7281)^a^ | | | | | | | | | | | | |
| <=0.86kg | 69 | 55.20 | 56 | 44.80 | 36.521 | 12.759 | 104.537 | **<.0001** | 1.810 | 0.202 | 16.248 | 0.7727 |
| 0.86-1kg | 123 | 90.44 | 13 | 9.56 | 4.756 | 1.515 | 14.929 | **0.0075** | 2.011 | 0.357 | 11.342 | 0.5013 |
| >1kg (ref) | 180 | 97.83 | 4 | 2.17 | 1 | x | x | x | 1 | x | x | x |
| Vitality score (p**<.0001**)^a^ | | | | | | | | | | | | |
| 0 (ref) | 296 | 98.67 | 4 | 1.33 | 1 | x | x | x | 1 | x | x | x |
| 1 | 74 | 71.15 | 30 | 28.85 | 30.000 | 10.250 | 87.805 | **<.0001** | 19.541 | 4.774 | 79.975 | 0.8564 |
| 2 | 2 | 4.88 | 39 | 95.12 | 1443.000 | 255.846 | 8138.690 | **<.0001** | 495.557 | 34.658 | >999.999 | **0.0001** |
| Intrauterine growth retardation score (p=0.3519)^a^ | | | | | | | | | | | | |
| 0 (ref) | 274 | 95.47 | 13 | 4.53 | 1 | x | x | x | 1 | x | x | x |
| 1 | 86 | 81.90 | 19 | 18.10 | 4.657 | 2.209 | 9.818 | **<.0001** | 1.798 | 0.380 | 8.514 | 0.8219 |
| 2 | 12 | 22.64 | 41 | 77.36 | 72.013 | 30.764 | 168.566 | **<.0001** | 4.173 | 0.567 | 30.684 | 0.1515 |
| Rectal temperature (p=0.0609)^a^ | | | | | | | | | | | | |
| ≤ 37.5 °C | 48 | 45.71 | 57 | 54.29 | 24.047 | 12.782 | 45.239 | **<.0001** | 2.986 | 0.951 | 9.370 | 0.0609 |
| > 37.5 °C (ref) | 324 | 95.29 | 16 | 4.71 | 1 | x | x | x | 1 | x | x | x |
| Sex (p=0.5040)^a^ | | | | | | | | | | | | |
| female (ref) | 187 | 84.23 | 35 | 15.77 | 1 | x | x | x | 1 | x | x |  |
| male | 185 | 82.96 | 38 | 17.04 | 1.097 | 0.664 | 1.813 | 0.7166 | 1.367 | 0.546 | 3.424 | 0.5040 |

^a^ General p-value Wald’s Chi²-Test; ^b^ p-value Wald’s Chi²-Test to the reference category
