# Supplementary material for: Killing underweighted low viable newborn piglets: Which health parameters are appropriate to make a decision?
Source: Porcine Health Manag. 2022 Jun 9;8:25. doi: 10.1186/s40813-022-00265-y (PMC9178864; doi:10.1186/s40813-022-00265-y)
Supplement: Supplementary file 2 — Additional file 2: Table 2. Logistic regression model for clinical variables related to piglets that spontaneously died until day 5 of age. Ref: reference category, OR: Odds Ratio Estimate, CI: confidence limits. p: level attained for the statistical test associated (aGeneral p-value Wald’s Chi²-Test; bp-value Wald’s Chi²-Test to the reference category). [file 40813_2022_265_MOESM2_ESM.docx]

Additional table 2: Logistic regression model for clinical variables related to piglets that **spontaneously died** until day 5 of age. ref: reference category, OR: Odds Ratio Estimate, CI: confidence limits. p: level attained for the statistical test associated

| Risk categories | alive | | spontaneously death | | univariable model | | | | multivariable model | | | |
| --- | --- | --- | --- | --- | --- | --- | --- | --- | --- | --- | --- | --- |
|  | n | % | n | % | OR | 95%-CI | | p | OR | 95%-CI | | p^b^ |
|  |  |  |  |  |  | low | up |  |  | low | up |  |
| Total | 372 |  | 74 |  | x |  |  | x | x |  |  | x |
| Herd/Group (p=**0.0294**)^a^ | | | | | | | | | | | | |
| 11(ref) | 26 | 65.00 | 14 | 35.00 | 1 | x | x | x | 1 | x | x | x |
| 12 | 27 | 87.10 | 4 | 12.90 | 0.275 | 0.080 | 0.946 | **0.0405** | 0.166 | 0.029 | 0.964 | 0.3252 |
| 13 | 19 | 79.17 | 5 | 20.83 | 0.489 | 0.150 | 1.591 | 0.2344 | 0.398 | 0.072 | 2.198 | 0.7922 |
| 21 | 33 | 86.84 | 5 | 13.16 | 0.281 | 0.090 | 0.883 | **0.0297** | 0.294 | 0.070 | 1.239 | 0.8280 |
| 22 | 25 | 83.33 | 5 | 16.67 | 0.371 | 0.117 | 1.184 | 0.0941 | 0.403 | 0.085 | 1.924 | 0.7395 |
| 23 | 38 | 84.44 | 7 | 15.56 | 0.342 | 0.121 | 0.963 | **0.0423** | 0.344 | 0.079 | 1.492 | 0.9476 |
| 31 | 26 | 86.67 | 4 | 13.33 | 0.286 | 0.083 | 0.984 | **0.0472** | 0.157 | 0.028 | 0.895 | 0.2707 |
| 32 | 35 | 87.50 | 5 | 12.50 | 0.265 | 0.085 | 0.830 | **0.0226** | 0.374 | 0.083 | 1.688 | 0.8335 |
| 33 | 38 | 95.00 | 2 | 5.00 | 0.098 | 0.020 | 0.467 | **0.0036** | 0.170 | 0.027 | 1.059 | 0.3708 |
| 41 | 43 | 87.76 | 6 | 12.24 | 0.259 | 0.089 | 0.758 | **0.0137** | 0.142 | 0.035 | 0.581 | 0.1205 |
| 42 | 28 | 73.68 | 10 | 26.32 | 0.663 | 0.251 | 1.752 | 0.4074 | 1.703 | 0.436 | 6.649 | **0.0005** |
| 43 | 34 | 82.93 | 7 | 17.07 | 0.382 | 0.135 | 1.083 | 0.0703 | 0.275 | 0.062 | 1.220 | 0.7256 |
| Body Weight (p=0.0598)^a^ | | | | | | | | | | | | |
| <=0.86kg | 69 | 61.61 | 43 | 38.39 | 16.023 | 6.879 | 37.325 | **<.0001** | 4.498 | 1.083 | 18.685 | 0.1123 |
| 0.86-1kg | 123 | 83.67 | 24 | 16.33 | 5.017 | 2.097 | 12.007 | **0.0003** | 3.166 | 1.165 | 8.608 | 0.2849 |
| >1kg (ref) | 180 | 96.26 | 7 | 3.74 | 1 | x | x | x | 1 | x | x | x |
| Vitality score (p=**0.0063**)^a^ | | | | | | | | | | | | |
| 0 (ref) | 296 | 91.93 | 26 | 8.07 | 1 | x | x | x | 1 | x | x | x |
| 1 | 74 | 69.81 | 32 | 30.19 | 4.923 | 2.766 | 8.765 | **<.0001** | 1.496 | 0.621 | 3.605 | **0.0328** |
| 2 | 2 | 11.11 | 16 | 88.89 | 91.062 | 19.845 | 417.847 | **<.0001** | 19.769 | 3.114 | 125.484 | **0.0016** |
| Intrauterine growth retardation score (p=0.1824)^a^ | | | | | | | | | | | | |
| 0 (ref) | 274 | 91.95 | 24 | 8.05 |  |  |  |  | 1 | x | x | x |
| 1 | 86 | 73.50 | 31 | 26.50 | 4.116 | 2.292 | 7.390 | **<.0001** | 1.684 | 0.610 | 4.653 | 0.6193 |
| 2 | 12 | 38.71 | 19 | 61.29 | 18.075 | 7.846 | 41.639 | **<.0001** | 4.207 | 0.902 | 19.627 | 0.0692 |
| Rectal temperature (p=**0.0002**)^a^ | | | | | | | | | | | | |
| ≤ 37.5 °C | 48 | 52.17 | 44 | 47.83 | 9.900 | 5.688 | 17.233 | **<.0001** | 4.181 | 1.949 | 8.967 | **0.0002** |
| > 37.5 °C (ref) | 324 | 91.53 | 30 | 8.47 | 1 | x | x | x | 1 | x | x | x |
| Sex (p=0.0573)^a^ | | | | | | | | | | | | |
| female (ref) | 187 | 85.78 | 31 | 14.22 | 1 | x | x | x | 1 | x | x | x |
| male | 185 | 81.14 | 43 | 18.86 | 1.402 | 0.847 | 2.322 | 0.1892 | 1.912 | 0.980 | 3.729 | 0.0573 |

^a^ General p-value Wald’s Chi²-Test; ^b^ p-value Wald’s Chi²-Test to the reference category
