# Supplementary material for: Killing underweighted low viable newborn piglets: Which health parameters are appropriate to make a decision?
Source: Porcine Health Manag. 2022 Jun 9;8:25. doi: 10.1186/s40813-022-00265-y (PMC9178864; doi:10.1186/s40813-022-00265-y)
Supplement: Supplementary file 3 — Additional file 3: Table 3. Logistic regression model for clinical and laboratory variables related to killing or death of newborn piglets dying until day 5 of age. Ref: reference category, OR: Odds Ratio Estimate, CI: confidence limits. p: level attained for the statistical test associated (aGeneral p-value Wald’s Chi²-Test; bp-value Wald’s Chi²-Test to the reference category). [file 40813_2022_265_MOESM3_ESM.docx]

Additional table 3: Logistic regression model for clinical and laboratory variables related to killing or death of newborn piglets dying until day 5 of age. ref: reference category, OR: Odds Ratio Estimate, CI: confidence limits. p: level attained for the statistical test associated

| Risk categories | alive | | dead | | univariable model | | | | multivariable model | | | |
| --- | --- | --- | --- | --- | --- | --- | --- | --- | --- | --- | --- | --- |
|  | n | % | n | % | OR | 95%-CI | | p | OR | 95%-CI | | p^b^ |
|  |  |  |  |  |  | low | up |  |  | low | up |  |
| Total | 372 | 71.68 | 147 | 28.32 | x | x | x | x | x | x | x | x |
| Herd/Group (p=**0.0069**) ^a^ | | | | | | | | | | | | |
| 11(ref) | 26 | 56.52 | 20 | 43.48 | 1 | x | x | x | 1 | x | x | x |
| 12 | 27 | 65.85 | 14 | 34.15 | 0.674 | 0.282 | 1.609 | 0.3741 | 0.272 | 0.061 | 1.214 | 0.6222 |
| 13 | 19 | 51.35 | 18 | 48.65 | 1.232 | 0.516 | 2.937 | 0.6386 | 0.691 | 0.163 | 2.936 | 0.2445 |
| 21 | 33 | 73.33 | 12 | 26.67 | 0.473 | 0.196 | 1.141 | 0.0956 | 0.382 | 0.098 | 1.496 | 0.9084 |
| 22 | 25 | 60.98 | 16 | 39.02 | 0.832 | 0.353 | 1.959 | 0.6738 | 0.795 | 0.219 | 2.885 | 0.0804 |
| 23 | 38 | 74.51 | 13 | 25.49 | 0.445 | 0.189 | 1.049 | 0.0643 | 0.382 | 0.099 | 1.478 | 0.9010 |
| 31 | 26 | 83.87 | 5 | 16.13 | 0.250 | 0.082 | 0.767 | **0.0153** | 0.086 | 0.015 | 0.502 | **0.0433** |
| 32 | 35 | 87.50 | 5 | 12.50 | 0.186 | 0.062 | 0.560 | **0.0028** | 0.240 | 0.054 | 1.072 | 0.4787 |
| 33 | 38 | 95.00 | 2 | 5.00 | 0.068 | 0.015 | 0.318 | **0.0006** | 0.085 | 0.013 | 0.543 | 0.0607 |
| 41 | 43 | 82.69 | 9 | 17.31 | 0.272 | 0.108 | 0.686 | **0.0058** | 0.182 | 0.051 | 0.645 | 0.1415 |
| 42 | 28 | 66.67 | 14 | 33.33 | 0.650 | 0.273 | 1.547 | 0.3301 | 1.460 | 0.405 | 5.266 | **0.0014** |
| 43 | 34 | 64.15 | 19 | 35.85 | 0.726 | 0.323 | 1.632 | 0.4390 | 0.453 | 0.130 | 1.576 | 0.5786 |
| Body Weight (p=0.0933) ^a^ | | | | | | | | | | | | |
| <=0.86kg | 69 | 41.07 | 99 | 58.93 | 23.475 | 11.870 | 46.424 | **<.0001** | 3.550 | 1.038 | 12.146 | 0.1040 |
| 0.86-1kg | 123 | 76.88 | 37 | 23.13 | 4.922 | 2.417 | 10.023 | **<.0001** | 2.450 | 1.001 | 5.998 | 0.4383 |
| >1kg (ref) | 180 | 94.24 | 11 | 5.76 | 1 | x | x | x | 1 | x | x | x |
| Vitality score (p**<.0001**) ^a^ | | | | | | | | | | | | |
| 0 (ref) | 296 | 90.80 | 30 | 9.20 | 1 | x | x | x | 1 | x | x | x |
| 1 | 74 | 54.41 | 62 | 45.59 | 8.265 | 4.989 | 13.694 | **<.0001** | 2.968 | 1.391 | 6.330 | 0.0994 |
| 2 | 2 | 3.51 | 55 | 96.49 | 271.333 | 63.013 | 1168.351 | **<.0001** | 43.399 | 7.298 | 258.098 | **0.0002** |
| Intrauterine growth retardation score (p=0.3331) ^a^ | | | | | | | | | | | | |
| 0 (ref) | 274 | 88.10 | 37 | 11.90 | 1 | x | x | x | 1 | x | x | x |
| 1 | 86 | 63.24 | 50 | 36.76 | 4.305 | 2.640 | 7.022 | **<.0001** | 1.311 | 0.535 | 3.210 | 0.5511 |
| 2 | 12 | 16.67 | 60 | 83.33 | 37.027 | 18.231 | 75.201 | **<.0001** | 2.605 | 0.693 | 9.788 | 0.1382 |
| Rectal temperature (p=**0.0032**) ^a^ | | | | | | | | | | | | |
| ≤ 37.5 °C | 48 | 32.21 | 101 | 67.79 | 14.818 | 9.336 | 23.519 | **<.0001** | 2.786 | 1.408 | 5.510 | **0.0032** |
| > 37.5 °C (ref) | 324 | 87.57 | 46 | 12.43 | 1 | x | x | x | 1 | x | x | x |
| Sex (p=0.0929) ^a^ | | | | | | | | | | | | |
| female (ref) | 187 | 73.91 | 66 | 26.09 | 1 | x | x | x | 1 | x | x |  |
| male | 185 | 69.55 | 81 | 30.45 | 1.241 | 0.846 | 1.820 | 0.2704 | 1.652 | 0.920 | 2.966 | 0.0929 |
| Immunocrit (p=0.0556) ^a^ | | | | | | | | | | | | |
| ≤ 0.1 | 79 | 43.89 | 101 | 56.11 | 8.143 | 5.307 | 12.494 | **<.0001** | 1.839 | 0.986 | 3.433 | 0.0556 |
| >0.1 (ref) | 293 | 86.43 | 46 | 13.57 | 1 | x | x | x | 1 | x | x | x |
| Glucose (p=0.1382) ^a^ | | | | | | | | | | | | |
| ≤ 70 | 159 | 56.99 | 120 | 43.01 | 5.954 | 3.739 | 9.481 | **<.0001** | 1.673 | 0.847 | 3.301 | 0.1382 |
| >70 (ref) | 213 | 88.75 | 27 | 11.25 | 1 | x | x | x | 1 | x | x | x |

^a^ General p-value Wald’s Chi²-Test; ^b^ p-value Wald’s Chi²-Test to the reference category
